# Is the Co-Occurrence of *Neophysopella meliosmae-myrianthae* and *N. montana* (*Pucciniales*) Common on Grapevines in Japan?

**DOI:** 10.3390/jof11030193

**Published:** 2025-03-03

**Authors:** Izumi Okane, Akiko Kurita, Yoshitaka Ono

**Affiliations:** 1 Institute of Life and Environmental Sciences, University of Tsukuba, Tsukuba 305-8572, Japan; 2 Agro-Bioresources Science and Technology, University of Tsukuba, Tsukuba 305-8572, Japan; kr1t.kk0@gmail.com; 3 College of Education, Ibaraki University, Mito 310-8512, Japan; yoshitaka.ono.grapes@vc.ibaraki.ac.jp

**Keywords:** rust fungi, grapevine leaf rust, *Vitis coignetiae*, DNA markers, co-infection

## Abstract

Grapevine leaf rust (GLR) in temperate Asia is caused by *Neophysopella meliosmae-myrianthae* and *N. montana*; the former is commonly found on commercial grape cultivars (*Vitis* spp.) and the latter on a wild grape species, *Vitis coignetiae*. The two GLR fungi were found to co-occur in *V. coignetiae* at two survey sites in Japan. Under experimental conditions, both fungi parasitize and develop into uredinial and telial stages on commercial grape cultivars and wild species. Despite the assumed involvement of *N. montana* in GLR symptoms in commercial vineyards, there has been no confirmed report of its incidence, and it is not clear whether *N. meliosmae-myrianthae* commonly occurs on *V. coignetiae* under natural conditions. In this study, we aimed to disclose the occurrence and, in particular, the co-occurrence of the two species in a wide array of commercial grape cultivars and *V. coignetiae* in Japan based on the detection of targeted DNA markers with specific PCR primer pairs. This study confirmed the occurrence of only *N. meliosmae-myrianthae* infection in symptomatic samples of grape cultivars, while the co-occurrence was observed only in *V. coignetiae*. *Neophysopella montana* was widely detected in *V. coignetiae* specimens.

## 1. Introduction

Five species are known to be involved in grapevine leaf rust (GLR): *Neophysopella meliosmae-myrianthae* (Henn. and Shirai) Jing X. Ji and Kakish.; *N. montana* (Y. Ono and Chatasiri) Jing X. Ji and Kakish.; *N. muscadiniae* (Buriticá) Jing X. Ji and Kakish.; *N. tropicalis* Y. Ono, Chatasiri, Pota and Okane; and *N. uva* (Buriticá and J.F. Hennen) Jing X. Ji and Kakish. (*Pucciniales*, *Basidiomycota*). *Neophysopella muscadiniae* and *N. uva* are distributed in the Americas, *N. tropicalis* in Southeast Asia [1,2,3], and *Neophysopella meliosmae-myrianthae* and *N. montana* in Japan [4]. Recently, *N. meliosmae-myrianthae* and *N. tropicalis* have invaded and firmly established in Brazil [1,5]. Temperate Asian grapevine leaf rust (GLR) is caused by *N. meliosmae-myrianthae* and *N. montana* [4,6], with the former commonly found on commercial grape cultivars (*Vitis* spp.) and the latter on a wild grape species, *Vitis coignetiae* Pulliat ex Planch. The two morphologically distinct fungi are different in their spermogonial–aecial host preference, i.e., the alternate hosts are *Meliosma myriantha* Siebold and Zucc. for the former fungus and *M. tenuis* Maxim. for the latter [4]. *Neophysopella meliosmae-myrianthae* has been reported to develop uredinial and telial stages on common commercial cultivars such as cv. ‘Delaware’ and cv. ‘Kyoho’ (*V. labruscana* L.H. Bailey), and wild species such as *V. coignetiae*, *V. thunbergii* Siebold and Zucc., and *V. flexuosa* Thunb. under natural conditions [6]. *Neophysopella montana* commonly occurs in *V. coignetiae* in the natural field. The two fungi were found to co-occur in wild *V. coignetiae* at two survey sites in Tochigi Prefecture, Japan [7]. In the inoculation experiments, both fungi parasitize and develop uredinial and telial stages on commercial grape cultivars, i.e., cv. Delaware and cv. Kyoho, in addition to wild species, i.e., *V. amurensis* Rupr., *V. coignetiae*, and *V. ficifolia* Bunge [4]. Despite the assumed involvement of *N. montana* in GLR diseases in commercial vineyards, there has been no confirmed report of its incidence. Similarly, it is not clear whether *N. meliosmae-myrianthae* commonly occurs on *V. coignetiae* under natural conditions [4,7].

Japan’s table grape production was 48,800 metric tons in 2023, ranking 16th in the world [8]. Japanese viticultural techniques, such as greenhouse forcing, seedless grape cultivation, and the development of various varieties have been uniquely developed, and the technology has been transferred overseas. Grape cultivation is an extremely important part of the Japanese fruit industry [9]. Determining how widespread the two GLR fungi are in both commercial vineyards and natural fields and at what frequencies they occur in diverse grape species is indispensable not only for developing practical and effective GLR management strategies for maintaining high grape quality and stable yields and supply but also for a better understanding of their biological nature and evolutionary relationships. In this study, we aimed to determine the occurrence of populations of the two GLR fungi in a wide array of commercial grape cultivars and the most common *V. coignetiae* in a whole geographic range of the main island, Honshu, Japan. While surveying a wide range of commercial vineyards in Japan to collect rust specimens for the detection of targeted fungal DNA markers, we also investigated the occurrence of *N. montana*, which had not previously been confirmed as a parasite of cultivated grapevines in commercial vineyards. We performed this investigation to verify its presence on wild grapevines in the vicinity of the surveyed areas of cultivated grapevine leaf rust.

## 2. Materials and Methods

Of the rust fungi parasitizing cultivated grape varieties, 34 specimens were collected by the authors, a total of 46 specimens were kept in the Mycological Herbarium of the University of Tsukuba (TSH) (formerly kept in the mycological herbarium of Ibaraki University (IBAR)), a total of two specimens were kept in the Faculty of Agriculture and Life Sciences of Hirosaki University (HHUF), and 21 specimens were provided by collaborators from governmental and prefectural agricultural research stations. In total, 103 specimens originating from 60 locations in 24 prefectures in Japan were examined (Table 1; Supplementary Table S1). The confirmed names of *Vitis* cultivars are listed in Table 1. The rust fungi parasitizing a wild grapevine, *V. coignetiae*, included eight specimens collected in this study, 23 specimens from the TSH (formerly kept in the IBAR), 11 preexistent specimens from TSH, and 2 specimens from the HHUF. In total, 44 specimens of rust fungi from wild grapevine leaves originating from fifteen locations in eight prefectures in Japan were examined (Table 2; Supplementary Table S2).

**Figure 1:**
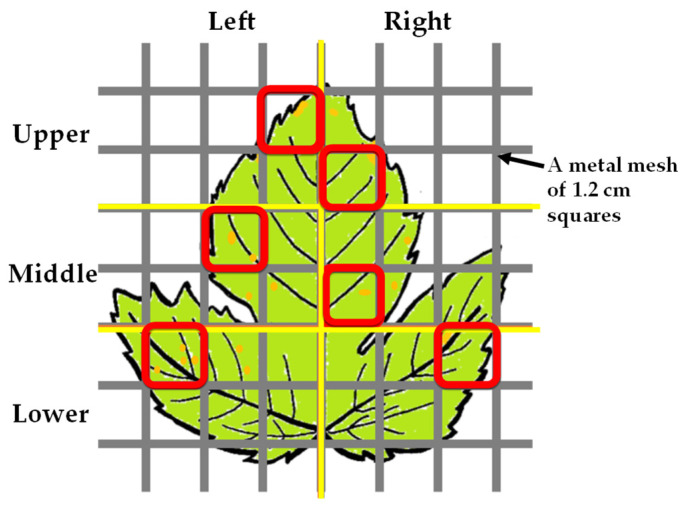
An example of selection of uredinia and telia for DNA extraction. A tested leaf was divided into 6 areas, separated by yellow lines (left, right, upper, middle, and lower), and a 1.2 cm square section (red box) where uredinia and/or telia (yellow spots) were present was selected in each area followed by spores sampling.

One leaf was arbitrarily selected from the specimens and was sectionalized into six areas, i.e., the left and right parts were further divided into upper, middle, and lower parts, using a metal mesh with square sections measuring approximately 1.2 cm (Figure 1). Spores were scraped from multiple uredinia or telia within a square section of the area using a surgical knife, and DNA extraction was performed. If no uredinium or telium was found in any square sections in the area, and the area was excluded from the analyses. In the case of the cultivated grapevines, DNA was extracted from the spores collected from 531 square sections of 103 specimens (Table 1), and in the case of the wild grapevines, DNA was extracted from spores collected from 166 square sections of 44 specimens (Table 2). DNA extraction was performed according to the methods described by Suyama et al. (1996) [10] and Virtudazo et al. (2001) [11]. In this study, we examined multiple DNA samples from one specimen because some of the specimens collected more than a decade ago were expected to degrade if subjected to this type of analysis.

**Figure 2:**
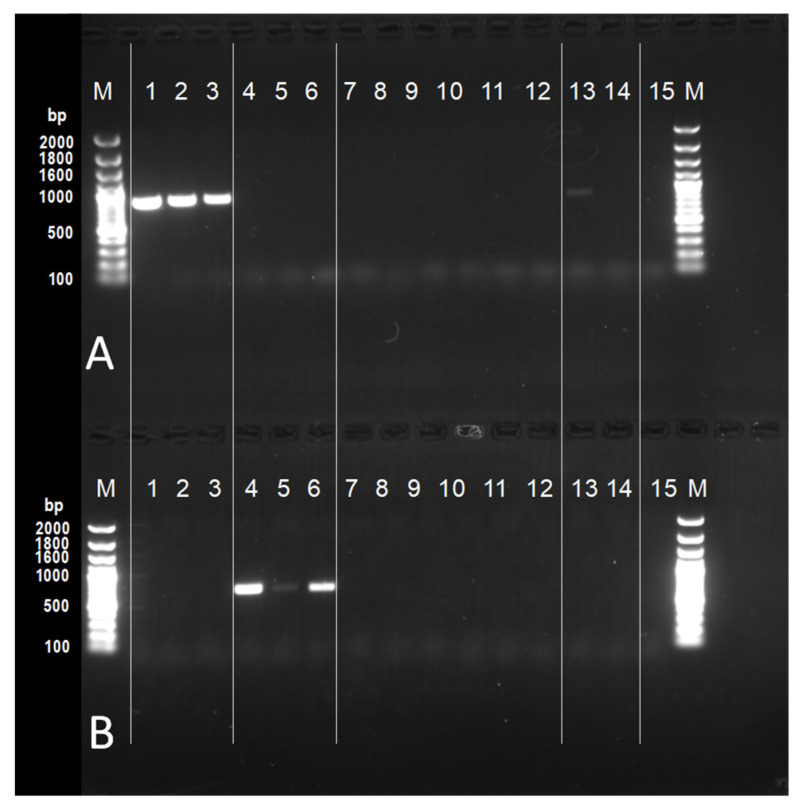
The result of the agarose-gel electrophoresis test for species-specific primers designed within the rDNA ITS 2 regions. (**A**) The test of the *Neophysopella meliosmae-myrianthae* specific-primer Pheuv1. (**B**) The test of the *N. montana* specific-primer PmontF. The primer NL4 (O’Donnell, 1993 [12]) was used as a reverse primer in both of the tests. 1–3: *N. meliosmae-myrianthae* (880 bp, IBAR 10494, 10485, and 10473, respectively), 4–6: *N. montana* (740 bp, IBAR 10455, 10463, and 10460, respectively), 7: *Pestalotiopsis* sp., 8: *Aspergillus* sp., 9: *Colletotrichum* sp.1, 10: *Colletotrichum* sp. 2, isolated from a fruit of grape, 11: *Plasmopara viticola*, 12: *Pestalotiopsis* sp. isolated from leaf of grapevine, 13: *N. ampelopsidis* (on *Ampelopsis glandulosa* var. *heterophylla*), 14: *N. vitis* (on *Parthenocissus tricuspidata*), 15: SDW, M: DNA marker.

The PCR of the extracted DNA was performed by using species-specific primers. The primers were designed within the rDNA ITS 2 regions based on the following sequence data, AB354778–AB354789 for *N. meliosmae-myrianthae*; KC815578–KC815584, KC815598 for *N. montana*, in this study (Supplementary Figure S1), and then these species’ specificity was rigorously confirmed in advance (Figure 2) to identify the rust fungi. A gradient PCR was performed to determine the optimal annealing temperature. PCR amplification was carried out in 0.2 mL microtubes with the following components: 12.5 µL of Emeral-dAmp^®^ MAX PCR Master Mix (2× Premix), 1.25 µL of *N. meliosmae-myrianthae*-specific primer Pheuv1 (5′-TGTTGCTGTTACTGGCTCAC-3′ (2 µM)), 1.25 µL of *N. montana*-specific primer PmontF (5′-CATTGATTACTCTGGTTTATTCCG-3′ (2 µM)), 2.5 µL of primer NL4 [12] (2 µM), 6.5 µL of sterile distilled water, and 1 µL of template DNA. The procedure was performed using a thermal cycler, following the same species-specific primer PCR amplification protocol: initial denaturation at 95 °C for 3 min, followed by 35 cycles of denaturation at 95 °C for 30 s, annealing at 60.7 °C for 1 min, extension at 72 °C for 1 min, and a final extension at 72 °C for 10 min.

Subsequently, the PCR products were electrophoresed on 1% agarose gel (containing 1× TAE buffer and SYBR Safe DNA Gel Stain, Thermo Fisher Scientific Inc., Waltham, MA, USA) in an electrophoresis tank filled with 1× TAE buffer for 25 min. Nippon Gene OneSTEP Ladder 100 (0.1–2 kbp) (Nippon Gene Co., Ltd., Tokyo, Japan) was used as a DNA size marker. The DNA sequences amplified with this specific method were used to identify the rust fungus/fungi that caused the foliar rust diseases.

## 3. Results

**Figure 3:**
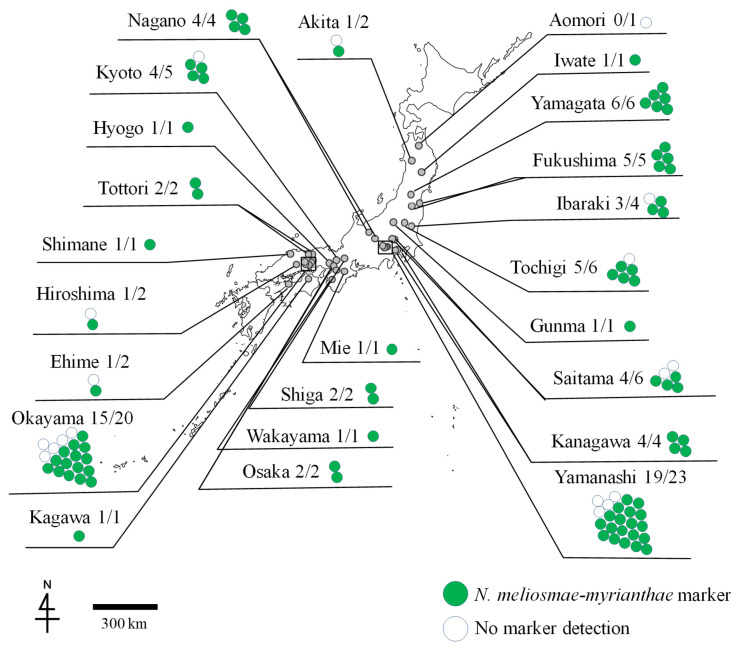
The distribution of DNA markers of *Neophysopella meliosmae-myrianthae* detected in the rust-infected specimens of *Vitis* cultivars in 24 prefectures of Japan. Numerals separated by a slash are the numbers of the specimens in which *N. meliosmae-myrianthae* markers were detected (left) and the total numbers of the rust-infected specimens examined (right). Colored circles indicate the result of DNA marker detection. *Neophysopella meliosmae-myrianthae* was detected, but *N. montana* was not.

As a result of the investigation of the rust infection in cultivated grapevines, *N. meliosmae-myrianthae* markers were detected in 395 DNA samples from 85 specimens collected at 53 locations in 23 prefectures out of 531 DNA samples from 103 specimens collected at 60 locations in 24 prefectures. No *N. montana* marker was detected in any of the samples (Table 1 and Figure 3). The remaining 135 DNA samples showed no amplification of the target DNA.

The samples that bore *N. meliosmae-myrianthae* sori included 22 specimens of cv. ‘Kyoho’, 8 of cv. ‘Pione’, 5 of cv. ‘Delaware’, and 3 specimens each of cv. ‘Fujiminori’ and cv. ‘Shine Muscat’, as well as one specimen each of cv. ‘Muscat Bailey A’, cv. ‘Queen Nina’, cv. ‘Oriental Star’, cv. ‘Black Beet’, cv. ‘Koshu’, cv. ‘Niagara’, cv. ‘Takatsuma’, cv. ‘Tenshu’, cv. ‘Takao’, cv. ‘Stuben’, cv. ‘Kai Noir’, and cv. ‘Yama Souvenir’.

**Figure 4:**
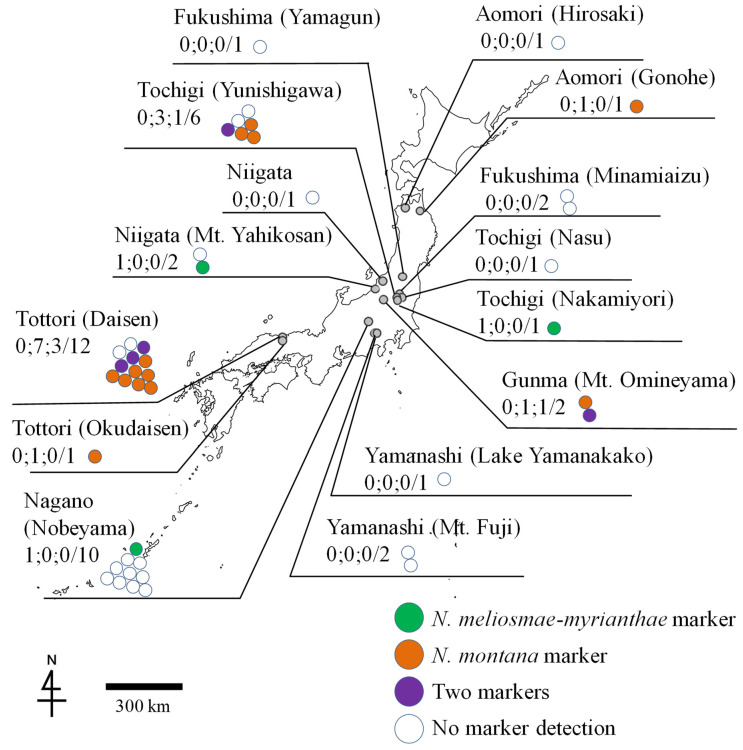
The distribution of DNA markers of *Neophysopella* species detected in the rust-infected specimens of *Vitis coignetiae* collected at 15 sites in eight prefectures of Japan. Numerals separated by semicolons are the numbers of specimens in which the DNA markers of *Neophysopella* species were detected: *N. meliosmae-myrianthae* markers (left), *N. montana* markers (center), and markers both of *N. meliosmae-myrianthae* and *N. montana* (right). The total number of the rust-infected specimens examined are shown on the left of a slash. Colored circles indicate the result of DNA marker detection.

As a result of the investigation of the rust infection in the wild grapevine *V. coignetiae*, out of 166 DNA samples from 44 specimens collected at 15 locations in 8 prefectures, infection with either *N. meliosmae-myrianthae* or *N. montana* or multiple infections in the same leaf were confirmed by the presence of 46 DNA markers in 20 specimens collected at seven locations in five prefectures (Table 2 and Figure 4). *Neophysopella montana*-only infection was confirmed by the presence of 31 DNA markers in thirteen specimens collected at a total of five locations in four prefectures, including 4 DNA markers in three specimens from one location in Tochigi Prefecture, 22 DNA markers in seven specimens from two locations in Tottori Prefecture, 4 DNA markers in two specimens from one location in Gunma Prefecture, and 1 DNA marker in one specimen from one location in Aomori Prefecture (Table 2 and Figure 4). Only *N. meliosmae-myrianthae* infection was confirmed by the presence of 9 DNA markers in three specimens collected at three locations in three prefectures, including 6 DNA markers in one specimen from one location in Niigata Prefecture, two in one specimen from one location in Tochigi Prefecture, and one in one specimen from one location in Nagano Prefecture (Table 2 and Figure 4). Multiple infections by both *N. montana* and *N. meliosmae-myrianthae* in the same leaf were confirmed in five specimens from three locations in three prefectures, including one specimen from one location in Tochigi Prefecture (TSH-R58372 = IBAR 10451), three specimens from one location in Tottori Prefecture (TSH-R58385 = IBAR 10464, TSH-R30536, and TSH-R30540), and one specimen from one location in Gunma Prefecture (TSH-R58429 = IBAR 10508) (Table 2 and Figure 4). In addition, 23 specimens showed no amplification of the target DNA. Most of these were collected in the 1900s (Table 2).

## 4. Discussion

In the inoculation experiments conducted by Ono et al. (2012) [4], *N. montana* was shown to infect two grape cultivars, ‘Kyoho’ and ‘Delaware’. Therefore, it was assumed that grapevine leaf rust on cultivated grapes, which had previously been reported to be caused by *N. meliosmae-myrianthae*, might also involve *N. montana* as an additional pathogen. To investigate this interesting phenomenon, we especially focused on GLR in cv. ‘Kyoho’ and cv. ‘Delaware’ in this study. Leaf samples of these cultivars were collected in areas near the distribution range of *M. tenuis*, the alternate host of *N. montana*, and tested with PCR analysis to confirm the presence of the rust fungus. As a result, only infections with *N. meliosmae-myrianthae* were confirmed in all the specimens of commercial grape cultivars tested.

Since Ono et al. (2012) [4] demonstrated that *N. montana* parasitizes a wild grape species, *V. coignetiae*, in natural environments, it was speculated that cv. ‘Yama Souvinion’, a grape cultivar derived from a cross between *V. coignetiae* and cv. ‘Cabernet Sauvignon’, might also be susceptible to *N. montana*. To investigate this, a survey was conducted in Fukushima Prefecture, especially in areas near the distribution range of *M. tenuis*. Leaf samples were collected from cv. ‘Yama-Souvinion’ plants showing symptoms of rust, and PCR analysis was performed. The results show that only *N. meliosmae-myrianthae* DNA was detected. The same approach was applied to cv. ‘Steuben’, cv. ‘Kai Noir’, and an unidentified grape cultivar (rootstock of cv. ‘Kai Noir’) collected at the same site as cv. ‘Yama Souvinion’. As a result, only *N. meliosmae-myrianthae* was detected.

A comprehensive study undertaken in Australia shows that almost all commercial grape cultivars are moderately to highly susceptible to GLR fungus (identified as *N. meliosmae-myrianthae*, now *N. tropicalis*) infection under conditions of an optimum temperature of 25 °C and saturated moisture for 6–12 h [13]. These study results are congruent with an early ecological study in *N. meliosmae-myrianthae* carried out in Japan [14]. No similar study has been undertaken for *N. montana*. From the inoculation experiments, however, it is assumed that both GLR fungi possess similar environmental requirements despite their different spermogonial–aecial preference. A possible reason why only *N. meliosmae-myrianthae* was found in human-managed vineyards might be a matter of geographic distance from a primary inoculum (spermogonial–aecial stages on *M. myriantha*).

The areas where *N. montana* was confirmed to occur in wild grapevines overlapped with the distribution of its alternate host, *M. tenuis* [13]; conversely, the areas where *N. meliosmae-myrianthae* was confirmed to occur overlapped with the distribution of *M. myriantha*. According to Horikawa’s distribution map (Supplementary Figure S2), the distribution of *M. tenuis* overlaps with that of *M. myriantha*, and both species coexist in some areas. The regions where PCR analysis confirmed multiple infections by both rust fungi (Omineyama, Gunma Prefecture; Yunishikawara, Tochigi Prefecture; Daisen, Tottori Prefecture) are considered to be areas where both rust fungi coexist and may potentially undergo host alternation, as both alternate hosts, *M. myriantha* and *M. tenuis*, have been reported (Supplementary Figure S1). In Nikko, Tochigi Prefecture, *N. montana* infections were found in four specimens of wild grapevines (including co-infections), and *N. meliosmae-myrianthae* infections were found in one specimen (co-infection). As a result of the testing of specimen TSH-R58372 (=IBAR 10451), PCR amplification products were obtained from two DNA samples, and *N. montana* DNA was detected in all samples. These results suggest that even in regions where both rust fungi are capable of host alternation, *N. montana* may have a greater tendency to cause leaf rust and is more likely to parasitize wild grapevines.

We demonstrated that the method using the species-specific primers developed in this study can also be used to detect such infections. While the co-infections of different rust species in wild grapevines were previously reported only in Tochigi Prefecture [7], this study confirmed such infections in Gunma Prefecture (one specimen from one location) and Tottori Prefecture (two specimens from one location). This suggests that co-infection may occur in regions beyond Tochigi Prefecture, as suggested by Ono (2016) [7], and that the coexistence of both rust fungi without clear spatial separation, i.e., without competition, may be possible within a single leaf.

In this study, we were unable to identify the most plausible cause(s) for the failure to detect target DNA in some rusted leaf samples, particularly from *V. coignetiae*. While this is in part likely due to contamination by other microbes or DNA degradation caused by a long storage period and adverse conditions, most of the specimens of GLR fungi of *V. coignetiae*, from which the target DNA markers were not amplified, were collected in higher-altitude and/or cold-climate areas, such as Nobeyama (ca. 1350 m ASL; Nagano Prefecture), and at the foot of Mt. Fuji and Lake Yamanakako (ca. 2000 m ASL and 1000 m ASL, respectively; Yamanashi Prefecture) (Table 2 and Figure 4). The possibility, therefore, that the rust populations represented by the samples studied were actually composed of cold-adapted variant subpopulations, having somewhat different DNA sequences that could not be detected with the primer pairs designed in this study, cannot be ruled out. These subpopulations may not necessarily represent a new taxon different from *N. montana* and *N. meliosmae-myrianthae*. Further studies would reveal the complex biodiversity of GLR fungi in the wild grape species, *V. coignetiae*, especially in higher mountainous areas in East Asia.

Contrarily to the results from the experimental inoculations, *N. meliosmae-myrianthae* was detected exclusively in commercial grape cultivars in all the geographic area surveyed, irrespective of their local climate or altitudinal differences. One of the possible causes of this selective occurrence of *N. meliosmae-myrianthae* on commercial grape cultivars might be the selective exclusion of *N. montana* by cultural practices to reduce diseases and pests. Major hosts of *N. montana* are wild grape species distributed away from commercial vineyards; thus, *N. montana* populations might be highly susceptible to fungicides, without having been selected for fungicide resistance.

Another possibility is the physical and/or ecological distance (or barriers) between commercial vineyards and the natural habitats of *M. tenuis*, the alternate host of *N. montana*. When in the uredinial stage, producing vegetative reproductive urediniospores, GLR fungi do not overwinter on grape canes or on dead grape leaves fallen on the ground [6]. The new infection cycle of *N. montana*, therefore, must start with the germination of teliospores in dead grape leaves fallen on the ground in the early spring through early summer. Basidiospores produced from the germination of overwintered teliospores subsequently infect and produce aeciospores on *M. tenuis*. The latter become the inoculum for a new rust fungus infection in grapevines, and the rust spreads from late summer on. Therefore, if *M. tenuis* is totally absent from the range of *V. coignetiae* or commercial grape cultivars, the incidence of *N. montana* rust is unlikely in that geographic range. The competitive exclusion of *N. montana* by *N. meliosmae-myrianthae* is not likely, and if it happens, it must be based on a sort of “first come, first served” principle [7]. Supposing that there is a time lag between the two fungi, in their arrival and successful infection in a single susceptible grape leaf, it is probable that the first-comer quickly occupies the susceptible host tissues to abundantly sporulate and that the late-comer cannot establish a new infection because of the little host tissue available. This competition assumes only physical occupation, but not physiological and biochemical relationships. Further experiments with different infection timings are needed to determine whether this sort of competition occurs in GLR. Contrarily, although not well documented in rust fungi, the co-occurrence of two or more rust species, in the same genus or different genera, in the same host individuals and even on the same leaves is a common phenomenon in rust fungi, as exemplified by Ono (2016) [7] and this study in GRL fungi. Ono et al. (2018) [15] explicitly showed that *Gymnoconia peckiana* (Howe) Trotter var. *verrucosa* (N. Zhang) J.Y. Zhuang and S.X. Wei, *Phragmidium sikangense* Petr., and *P. barclayi* Dietel co-occur in close proximity on a leaf of *Rubus erythrocarpus* T.T. Yu and L.T. Lu. The co-occurrence or mixed infection of two or more plant pathogens (not restricted to fungal pathogens) is frequently observed, and its synergistic effects on disease incidence and development are widely acknowledged [16,17]. For this reason, any changes in environmental conditions and agricultural management, that might encourage the encounter of the two *Neophysopella* fungi in intensive vineyards, would facilitate sever GLR incidence.

## Figures and Tables

**Table 1:** List of the cultivated grapevine samples tested and the result of the DNA detection of the two targeted grapevine leaf rust fungi, *Neophysopella meliosmae-myrianthae* and *N. montana*.

Specimen Number	Location	Grapevine Cultivar (*V. ×*: Hybridization with *V. vinifera*)	Detection of *N. meliosmae-myrianthae* (No. of Target DNA Samples Detected/Total No. of DNA Samples)	Detection of *N. montana* (No. of Target DNA Samples Detected/Total No. of DNA Samples)
TSH-R58051 (=IBAR 10118)	Arakawa, Chichibu City, Saitama Pref.	Unknown	Negative (0/6)	Negative (0/6)
TSH-R58052 (=IBAR 10119)	Arakawa, Chichibu City, Saitama Pref.	Unknown	Negative (0/4)	Negative (0/4)
TSH-R58171 (=IBAR 10241)	Hiroshima Agricultural Technology Center, Higashihiroshima City, Hiroshima Pref.	*V. vinifera × V. labrascana* ‘Shine Muscat’	Positive (2/3)	Negative (0/3)
TSH-R58172 (=IBAR 10242)	Hiroshima Agricultural Technology Center, Higashihiroshima City, Hiroshima Pref.	*V. × labruscana* ‘Muscat Bailey A’	Negative (0/3)	Negative (0/3)
TSH-R58387 (=IBAR 10466)	Hojo, Hokuei Town, Tohaku, Tottori Pref.	*V. × labruscana* ‘Kyoho’	Positive (6/6)	Negative (0/6)
TSH-R58388 (=IBAR 10467)	Kose, Mimasaka City, Okayama Pref.	Unknown	Negative (0/6)	Negative (0/6)
TSH-R58389 (=IBAR 10468)	Kose, Mimasaka City, Okayama Pref.	Unknown	Negative (0/5)	Negative (0/5)
TSH-R58390 (=IBAR 10469)	Kose, Mimasaka City, Okayama Pref.	Unknown	Positive (6/6)	Negative (0/6)
TSH-R58391 (=IBAR 10470)	Kose, Mimasaka City, Okayama Pref.	Unknown	Positive (4/6)	Negative (0/6)
TSH-R58392 (=IBAR 10471)	Saeki Town, Wake, Okayama Pref.	Unknown	Negative (0/5)	Negative (0/5)
TSH-R58393 (=IBAR 10472)	Saeki Town, Wake, Okayama Pref.	Unknown	Positive (4/5)	Negative (0/5)
TSH-R58394 (=IBAR 10473)	Saeki Town, Wake, Okayama Pref.	Unknown	Positive (6/6)	Negative (0/6)
TSH-R58395 (=IBAR 10474)	Saeki Town, Wake, Okayama Pref.	Unknown	Positive (6/6)	Negative (0/6)
TSH-R58397 (=IBAR 10476)	National Route 429, Kita City, Okayama Pref.	Unknown	Negative (0/6)	Negative (0/6)
TSH-R58398 (=IBAR 10477)	Prefectural Route 66, Katta, Maniwa City, Okayama Pref.	Unknown	Positive (4/5)	Negative (0/5)
TSH-R58399 (=IBAR 10478)	Yoshikawa, Kibichuo Town, Kaga, Okayama Pref.	Unknown	Positive (3/3)	Negative (0/3)
TSH-R58400 (=IBAR 10479)	Yoshikawa, Kibichuo Town, Kaga, Okayama Pref.	Unknown	Positive (6/6)	Negative (0/6)
TSH-R58401 (=IBAR 10480)	Yoshikawa, Kibichuo Town, Kaga, Okayama Pref.	Unknown	Positive (6/6)	Negative (0/6)
TSH-R58402 (=IBAR 10481)	Shirochi, Ochiai Town, Takahashi City, Okayama Pref.	Unknown	Positive (6/6)	Negative (0/6)
TSH-R58403 (=IBAR 10482)	Higashikarube, Akaiwa City, Okayama Pref.	Unknown	Negative (0/6)	Negative (0/6)
TSH-R58404 (=IBAR 10483)	Higashikarube, Akaiwa City, Okayama Pref.	Unknown	Positive (2/2)	Negative (0/2)
TSH-R58405 (=IBAR 10484)	Ashigakubo, Yokoze Town, Chichibu, Saitama Pref.	*V. × labruscana* ‘Kyoho’	Positive (6/6)	Negative (0/6)
TSH-R58406 (=IBAR 10485)	Ashigakubo, Yokoze Town, Chichibu, Saitama Pref.	*V. × labruscana* ‘Kyoho’	Positive (6/6)	Negative (0/6)
TSH-R58407 (=IBAR 10486)	Ashigakubo, Yokoze Town, Chichibu, Saitama Pref.	*V. × labruscana* ‘Kyoho’	Positive (6/6)	Negative (0/6)
TSH-R58408 (=IBAR 10487)	Yokoze, Yokoze Town, Chichibu, Saitama Pref.	*V. × labruscana* ‘Kyoho’	Positive (6/6)	Negative (0/6)
TSH-R58409 (=IBAR 10488)	Makiokacho Kurashina, Yamanashi City, Yamanashi Pref.	*V. × labruscana* ‘Kyoho’	Positive (6/6)	Negative (0/6)
TSH-R58410 (=IBAR 10489)	Higashi, Yamanashi City, Yamanashi Pref.	Unknown	Positive (5/6)	Negative (0/6)
TSH-R58411 (=IBAR 10490)	Ochiai, Yamanashi City, Yamanashi Pref.	Unknown	Positive (6/6)	Negative (0/6)
TSH-R58412 (=IBAR 10491)	Kasugai, Fuefuki City, Yamanashi Pref.	Unknown	Negative (0/6)	Negative (0/6)
TSH-R58413 (=IBAR 10492)	Sakurai, Fuefuki City, Yamanashi Pref.	Unknown	Positive (6/6)	Negative (0/6)
TSH-R58414 (=IBAR 10493)	Isawa Town, Fuefuki City, Yamanashi Pref.	Unknown	Negative (0/6)	Negative (0/6)
TSH-R58415 (=IBAR 10494)	Enzan Kaminishi, Koshu City, Yamanashi Pref.	Unknown	Positive (6/6)	Negative (0/6)
TSH-R58416 (=IBAR 10495)	Enzan Kamioso, Koshu City, Yamanashi Pref.	Unknown	Positive (6/6)	Negative (0/6)
TSH-R58417 (=IBAR 10496)	Yama, Katsunuma Town, Koshu City, Yamanashi Pref.	Unknown	Negative (0/6)	Negative (0/6)
TSH-R58418 (=IBAR 10497)	Osade, Katsunuma Town, Koshu City, Yamanashi Pref.	Unknown	Positive (6/6)	Negative (0/6)
TSH-R58419 (=IBAR 10498)	Katsunuma, Katsunuma Town, Koshu City, Yamanashi Pref.	Unknown	Positive (6/6)	Negative (0/6)
TSH-R58420 (=IBAR 10499)	Katsunuma, Katsunuma Town, Koshu City, Yamanashi Pref.	*V. × labruscana* ‘Pione’	Positive (6/6)	Negative (0/6)
TSH-R58421 (=IBAR 10500)	Kamiyama Town, Nirasaki City, Yamanashi Pref.	Unknown	Positive (3/3)	Negative (0/3)
TSH-R58422 (=IBAR 10501)	Kamiyama Town, Nirasaki City, Yamanashi Pref.	Unknown	Positive (4/4)	Negative (0/4)
TSH-R58427 (=IBAR 10506)	Kamishiroi, Shibukawa City, Gunma Pref.	Unknown	Positive (1/3)	Negative (0/3)
TSH-R58430 (=IBAR 10509)	Shimotsubara, Iwafune Town, Shimotsuga, Tochigi Pref.	*V. × labruscana* ‘Kyoho’	Positive (6/6)	Negative (0/6)
TSH-R58431 (=IBAR 10510)	Shimotsubara, Iwafune Town, Shimotsuga, Tochigi Pref.	*V. × labruscana* ‘Kyoho’	Positive (6/6)	Negative (0/6)
TSH-R58432 (=IBAR 10511)	Shimotsubara, Iwafune Town, Shimotsuga, Tochigi Pref.	*V. × labruscana* ‘Kyoho’	Positive (5/6)	Negative (0/6)
TSH-R58433 (=IBAR 10512)	Nishiyamada, Oohira Town, Tochigi City, Tochigi Pref.	*V. × labruscana* ‘Kyoho’	Positive (6/6)	Negative (0/6)
TSH-R58434 (=IBAR 10513)	Nishiyamada, Oohira Town, Tochigi City, Tochigi Pref.	*V. × labruscana* ‘Kyoho’	Negative (0/6)	Negative (0/6)
TSH-R58435 (=IBAR 10514)	Nishiyamada, Oohira Town, Tochigi City, Tochigi Pref.	*V. × labruscana* ‘Kyoho’	Positive (6/6)	Negative (0/6)
TSH-R30450	Ezohara, Yamanashi City, Yamanashi Pref.	*V. × labruscana* ‘Kyoho’	Positive (6/6)	Negative (0/6)
TSH-R30451	Nanokaichiba, Yamanashi City, Yamanashi Pref.	*V. × labruscana* ‘Kyoho’	Positive (6/6)	Negative (0/6)
TSH-R30453	Oyama, Misaka Town, Fuefuki City, Yamanashi Pref.	*V. × labruscana* ‘Kyoho’	Positive (5/5)	Negative (0/5)
TSH-R30455	Katsunuma Town, Koshu City, Yamanashi Pref.	*V. vinifera × V. labrascana* ‘Queen Nina’	Negative (0/6)	Negative (0/6)
TSH-R30456	Nanokaichiba, Yamanashi City, Yamanashi Pref.	*V. vinifera × V. labrascana* ‘Oriental Star’	Positive (6/6)	Negative (0/6)
TSH-R30457	Manriki, Yamanashi City, Yamanashi Pref.	*V. × labruscana* ‘Kyoho’	Positive (6/6)	Negative (0/6)
TSH-R30461	Shimoidai Town, Matsuyama City, Ehime Pref.	*V. × labruscana* ‘Fujiminori’	Negative (0/6)	Negative (0/6)
TSH-R30462	Shimoidai Town, Matsuyama City, Ehime Pref.	*V. × labruscana* ‘Black Beet’	Positive (6/6)	Negative (0/6)
TSH-R30463	Fruit-Tree Experiment Station, Tenno, Katagami City, Akita Pref.	*V. × labruscana* ‘Campbell Early’?	Negative (0/6)	Negative (0/6)
TSH-R30464	Fruit-Tree Experiment Station, Tenno, Katagami City, Akita Pref.	*V. × labruscana* ‘Black Beet’?	Positive (6/6)	Negative (0/6)
TSH-R30466	Kuroki, Soma City, Fukushima Pref.	*V. vinifera × V. labrascana* ‘Shine Muscat’?	Positive (6/6)	Negative (0/6)
TSH-R30467	Yokone Town, Kofu City, Yamanashi Pref.	*V. × labruscana* ‘Delaware’	Positive (6/6)	Negative (0/6)
TSH-R30468	Yokone Town, Kofu City, Yamanashi Pref.	*V. vinifera* ‘Koshu’ or *V.* × *labruscana* ‘Pione’	Positive (6/6)	Negative (0/6)
TSH-R30469	Yokone Town, Kofu City, Yamanashi Pref.	*V. vinifera* ‘Koshu’	Positive (6/6)	Negative (0/6)
TSH-R30470	Pre. Res. Ins. for the Environ., Agricul., For. and Fisher., Habikino City, Osaka Pref.	*V. × labruscana* ‘Delaware’	Positive (6/6)	Negative (0/6)
TSH-R30472	Akaiwa City, Okayama Pref.	*V. × labruscana* ‘Pione’	Positive (6/6)	Negative (0/6)
TSH-R30473	Aono, Ibara City, Okayama Pref.	*V. × labruscana* ‘Pione’	Positive (6/6)	Negative (0/6)
TSH-R30475	Tsuchida, Okayama City, Okayama Pref.	*V. × labruscana* ‘Pione’	Positive (6/6)	Negative (0/6)
TSH-R30476	Nagasaki, Funao Town, Kurashiki City, Okayama Pref.	*V. × labruscana* ‘Pione’	Positive (5/6)	Negative (0/6)
TSH-R30477	Izumo City, Shimane Pref.	*V. × labruscana* ‘Delaware’	Positive (2/2)	Negative (0/2)
HHUF 10428	Sakaimatsu, Kuroishi City, Aomori Pref.	Unknown	Negative (0/6)	Negative (0/6)
HHUF 15175	Oohasama Town, Hanamaki City, Iwate Pref.	Unknown	Positive (2/6)	Negative (0/6)
TSH-R30478	Hashimoto, Yazu Town, Yazu, Tottori Pref.	Unknown	Positive (5/6)	Negative (0/6)
TSH-R30517	Atsugi City, Kanagawa Pref.	Unknown	Positive (6/6)	Negative (0/6)
TSH-R30480	Kita Town, Kobe City, Hyogo Pref.	*V. × labruscana* ‘Pione’	Positive (1/1)	Negative (0/1)
TSH-R30482	Kyoto City, Kyoto Pref.	*V. vinifera × V. labrascana* ‘Shine Muscat’	Negative (0/5)	Negative (0/5)
TSH-R30483	Kyoto City, Kyoto Pref.	*V. × labruscana* ‘Delaware’	Positive (4/4)	Negative (0/4)
TSH-R30484	Kyoto City, Kyoto Pref.	*V. × labruscana* ‘Pione’	Positive (3/3)	Negative (0/3)
TSH-R30485	Kyoto City, Kyoto Pref.	*V. × labruscana* ‘Fujiminori’	Positive (4/5)	Negative (0/5)
TSH-R30487	Ryuo Town, Shiga Pref.	*V. × labruscana* ‘Kyoho’	Positive (4/4)	Negative (0/4)
TSH-R30488	Ryuo Town, Shiga Pref.	*V. × labruscana* ‘Kyoho’	Positive (1/2)	Negative (0/2)
TSH-R30489	Nabari City, Mie Pref.	*V. × labruscana* ‘Kyoho’	Positive (5/5)	Negative (0/5)
TSH-R30491	Kashiwara City, Osaka Pref.	*V. × labruscana* ‘Delaware’	Positive (5/5)	Negative (0/5)
TSH-R30493	Aridagawa Town, Wakayama Pref.	*V. × labruscana* ‘Kyoho’	Positive (4/4)	Negative (0/4)
TSH-R30495	Okaya City, Nagano Pref.	*V. × labruscana* ‘Kyoho’	Positive (1/1)	Negative (0/1)
TSH-R30496	Okaya City, Nagano Pref.	*V. × labrusca* ‘Niagara’	Positive (1/2)	Negative (0/2)
TSH-R30497	Ikusaka Village, Higashichikuma, Nagano Pref.	*V. vinifera × V. labrascana* ‘Shine Muscat’	Positive (1/1)	Negative (0/1)
TSH-R30499	Ikusaka Village, Higashichikuma, Nagano Pref.	*V. × labruscana* ‘Kyoho’	Positive (1/2)	Negative (0/2)
TSH-R30505	Kasumigaura City, Ibaraki Pref.	*V. × labruscana* ‘Kyoho’	Negative (0/5)	Negative (0/5)
TSH-R30507	Kasumigaura City, Ibaraki Pref.	*V. × labruscana* ‘Kyoho’	Positive (6/6)	Negative (0/6)
TSH-R30515	Kasumigaura City, Ibaraki Pref.	*V. × labruscana* ‘Muscat Bailey A’	Positive (6/6)	Negative (0/6)
TSH-R30516	Kasumigaura City, Ibaraki Pref.	*V. × labruscana* ‘Takatsuma’	Positive (4/6)	Negative (0/6)
TSH-R30518	Tokyo Univ. of Agricul. and Tech. Isehara Farm, Sannomiya, Isehara City, Kanagawa Pref.	*V. × labruscana* ‘Fujiminori’	Positive (6/6)	Negative (0/6)
TSH-R30519	Tokyo Univ. of Agricul. and Tech. Isehara Farm, Sannomiya, Isehara City, Kanagawa Pref.	*V. vinifera × V. labrascana* ‘Shine Muscat’	Positive (2/2)	Negative (0/2)
TSH-R30520	Tokyo Univ. of Agricul. and Tech. Isehara Farm, Sannomiya, Isehara City, Kanagawa Pref.	*V. vinifera × V. labrascana* ‘Queen Nina’	Positive (3/6)	Negative (0/6)
TSH-R30521	Kaminoyama City, Yamagata Pref.	*V. × labruscana* ‘Kyoho’?	Positive (3/5)	Negative (0/5)
TSH-R30522	Kaminoyama City, Yamagata Pref.	*V. × labruscana* ‘Pione’	Positive (5/5)	Negative (0/5)
TSH-R30523	Kaminoyama City, Yamagata Pref.	*V. × labruscana* ‘Tenshu’	Positive (3/4)	Negative (0/4)
TSH-R30526	Kaminoyama City, Yamagata Pref.	*V. × labruscana* ‘Fujiminori’	Positive (3/5)	Negative (0/5)
TSH-R30527	Kaminoyama City, Yamagata Pref.	*V. × labruscana* ‘Takao’	Positive (5/5)	Negative (0/5)
TSH-R30530	Kaminoyama City, Yamagata Pref.	Unknown	Positive (4/6)	Negative (0/6)
TSH-R30531	Nihonmatsu City, Fukushima Pref.	*V. coignetiae × V. vinifera* ‘Yama Sauvignon’	Positive (2/2)	Negative (0/2)
TSH-R30532	Nihonmatsu City, Fukushima Pref.	*V. × labruscana* ‘Steuben’	Positive (6/6)	Negative (0/6)
TSH-R30533	Nihonmatsu City, Fukushima Pref.	*V. × labruscana* ‘Kai Noir’	Positive (1/6)	Negative (0/6)
TSH-R30534	Nihonmatsu City, Fukushima Pref.	Unknown	Positive (6/6)	Negative (0/6)
TSH-R30486	Kyoto City, Kyoto Pref.	Unknown	Positive (6/6)	Negative (0/6)
TSH-R30543	Takamatsu City, Kagawa Pref.	Unknown	Positive (5/6)	Negative (0/6)

HHUF: The Mycological Herbarium of Hirosaki University, IBAR: The Herbarium of Systematic Mycology, Ibaraki University, TSH-R: The Rust Collection of Mycological Herbarium of the University of Tsukuba.

**Table 2:** List of wild grapevine, *Vitis coignetiae*, samples tested and the result of the DNA detection of the two targeted grapevine leaf rust fungi, *Neophysopella meliosmae-myrianthae* and *N. montana*.

Specimen Number	Location	Detection of *N. meliosmae-myrianthae* (No. of Target DNA Samples Detected/Total No. of DNA Samples)	Detection of *N. montana* (No. of Target DNA Samples Detected/Total No. of DNA Samples)
TSH-R51432 (=IBAR 1817)	Mt. Owasezawasan, Yama, Fukushima Pref.	Negative (0/6)	Negative (0/6)
TSH-R52161 (=IBAR 2782)	Takizawa forest road, Mt. Fuji, Yamanashi Pref.	Negative (0/4)	Negative (0/4)
TSH-R52162 (=IBAR 2783)	Takizawa forest road, Mt. Fuji, Yamanashi Pref.	Negative (0/6)	Negative (0/6)
TSH-R53179 (=IBAR 3930)	Nasu Town, Nasu, Tochigi Pref.	Negative (0/6)	Negative (0/6)
TSH-R54801 (=IBAR 6279)	Mt. Yahikoyama, Yahiko Village, Nishikanbara, Niigata Pref.	Negative (0/6)	Negative (0/6)
TSH-R54804 (=IBAR 6282)	Mt. Yahikoyama, Yahiko Village, Nishikanbara, Niigata Pref.	Positive (6/6)	Negative (0/6)
TSH-R58370 (=IBAR 10449)	Nakamiyori, Nikko City, Tochigi Pref.	Positive (2/2)	Negative (0/2)
TSH-R58371 (=IBAR 10450)	Yunishigawa, Nikko City, Tochigi Pref.	Negative (0/4)	Positive (1/4)
TSH-R58372 (=IBAR 10451)	Yunishigawa, Nikko City, Tochigi Pref.	Positive (1/6)	Positive (2/6)
TSH-R58373 (=IBAR 10452)	Yunishigawa, Nikko City, Tochigi Pref.	Negative (0/4)	Positive (1/4)
TSH-R58374 (=IBAR 10453)	Yunishigawa, Nikko City, Tochigi Pref.	Negative (0/6)	Negative (0/6)
TSH-R58375 (=IBAR 10454)	Yunishigawa, Nikko City, Tochigi Pref.	Negative (0/6)	Negative (0/6)
TSH-R58376 (=IBAR 10455)	Yunishigawa, Nikko City, Tochigi Pref.	Negative (0/3)	Positive (2/3)
TSH-R58377 (=IBAR 10456)	Yasugamori Forest Road, Minamiaizu, Fukushima Pref.	Negative (0/6)	Negative (0/6)
TSH-R58378 (=IBAR 10457)	Yasugamori Forest Road, Minamiaizu, Fukushima Pref.	Negative (0/5)	Negative (0/5)
TSH-R58379 (=IBAR 10458)	Mt. Daisen, Daisen Town, Saihaku, Tottori Pref.	Negative (0/4)	Positive (2/4)
TSH-R58380 (=IBAR 10459)	Mt. Daisen, Daisen Town, Saihaku, Tottori Pref.	Negative (0/6)	Negative (0/6)
TSH-R58381 (=IBAR 10460)	Mt. Daisen, Daisen Town, Saihaku, Tottori Pref.	Negative (0/6)	Positive (6/6)
TSH-R58384 (=IBAR 10463)	Mt. Daisen, Daisen Town, Saihaku, Tottori Pref.	Negative (0/5)	Positive (3/5)
TSH-R58385 (=IBAR 10464)	Mt. Daisen, Daisen Town, Saihaku, Tottori Pref.	Positive (1/5)	Positive (2/5)
TSH-R58386 (=IBAR 10465)	Okudaisen, Kofu Town, Hino, Tottori Pref.	Negative (0/6)	Positive (2/6)
TSH-R58428 (=IBAR 10507)	Mt. Omineyama, Minakami Town, Tone, Gunma Pref.	Negative (0/6)	Positive (3/6)
TSH-R58429 (=IBAR 10508)	Mt. Omineyama, Minakami Town, Tone, Gunma Pref.	Positive (1/2)	Positive (1/2)
HHUF 3963	Hengasa Forest Road, Hirosaki City, Aomori Pref.	Negative (0/3)	Negative (0/3)
HHUF 11069	Jizotai, Gonohe Town, Sannohe, Aomori Pref.	Negative (0/6)	Positive (1/6)
TSH-R30536	Mt. Daisen, Daisen Town, Saihaku, Tottori Pref.	Negative (0/1)	Negative (0/1)
TSH-R30537	Mt. Daisen, Daisen Town, Saihaku, Tottori Pref.	Negative (0/1)	Positive (1/1)
TSH-R30538	Mt. Daisen, Daisen Town, Saihaku, Tottori Pref.	Positive (1/3)	Positive (2/3)
TSH-R30539	Mt. Daisen, Daisen Town, Saihaku, Tottori Pref.	Negative (0/5)	Positive (5/5)
TSH-R30540	Mt. Daisen, Daisen Town, Saihaku, Tottori Pref.	Positive (1/1)	Positive (1/1)
TSH-R30541	Mt. Daisen, Daisen Town, Saihaku, Tottori Pref.	Negative (0/2)	Positive (2/2)
TSH-R30542	Mt. Daisen, Daisen Town, Saihaku, Tottori Pref.	Negative (0/1)	Positive (1/1)
TSH-R2204	Nobeyama, Minamimaki Village, Minamisaku, Nagano Pref.	Negative (0/1)	Negative (0/1)
TSH-R2205	Nobeyama, Minamimaki Village, Minamisaku, Nagano Pref.	Negative (0/1)	Negative (0/1)
TSH-R2206	Nobeyama, Minamimaki Village, Minamisaku, Nagano Pref.	Negative (0/1)	Negative (0/1)
TSH-R2207	Nobeyama, Minamimaki Village, Minamisaku, Nagano Pref.	Negative (0/1)	Negative (0/1)
TSH-R2803	Nobeyama, Minamimaki Village, Minamisaku, Nagano Pref.	Negative (0/1)	Negative (0/1)
TSH-R2804	Nobeyama, Minamimaki Village, Minamisaku, Nagano Pref.	Negative (0/1)	Negative (0/1)
TSH-R2805	Nobeyama, Minamimaki Village, Minamisaku, Nagano Pref.	Negative (0/1)	Negative (0/1)
TSH-R2806	Nobeyama, Minamimaki Village, Minamisaku, Nagano Pref.	Negative (0/1)	Negative (0/1)
TSH-R3452	Nobeyama, Minamimaki Village, Minamisaku, Nagano Pref.	Negative (0/5)	Negative (0/5)
TSH-R13225	Lake Yamanakako, Yamanashi Pref.	Negative (0/4)	Negative (0/4)
TSH-R1606	Niigata Pref.	Negative (0/6)	Negative (0/6)
TSH-R30544	Nobeyama, Minamimaki Village, Minamisaku, Nagano Pref.	Positive (4/4)	Negative (0/4)

HHUF: The Mycological Herbarium of Hirosaki University, IBAR: The Herbarium of Systematic Mycology, Ibaraki University, TSH-R: The Rust Collection of Mycological Herbarium of the University of Tsukuba.

## Data Availability

The original contributions of this study are included in the article/Supplementary Materials, and further inquiries can be directed to the corresponding author.

## Supplementary Materials

The following supporting information can be downloaded at https://www.mdpi.com/article/10.3390/jof11030193/s1, Figure S1: Sequence alignment of the rDNA ITS2 regions for the design of species-specific primers for *Neophysopella meliosmae-myriantha* and *N. montana*. Figure S2: Distribution map of *Meliosma myriantha* (left) and *M. tenuis* (right) in Japan (Horikawa, 1972 [18]). Table S1: List of location, geographical coordinate, and collection date of cultivated grapevine samples tested. Table S2: List of locality, geographical coordinate, and collection date of wild grapevine, *Vitis coignetiae*, samples tested.
