# Supplementary material for: Is the Co-Occurrence of Neophysopella meliosmae-myrianthae and N. montana (Pucciniales) Common on Grapevines in Japan?
Source: J Fungi (Basel). 2025 Mar 3;11(3):193. doi: 10.3390/jof11030193 (PMC11943129; doi:10.3390/jof11030193)
Supplement: Supplementary file 1 [file jof-11-00193-s001.zip › jof-3415858-supplementary/Supplementary Figure S1.pdf]

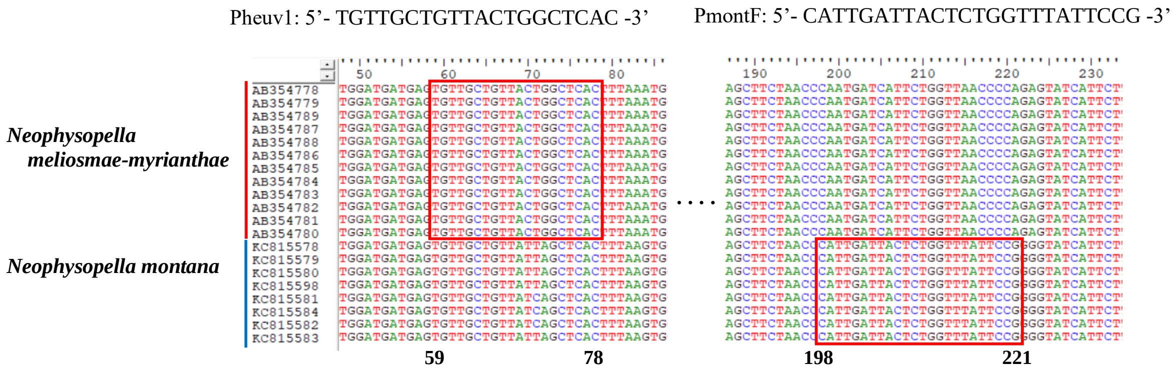

Supplementary Figure S1. Sequence alignment of the rDNA ITS2 regions for the design of species-specific primers for *Neophysopella meliosmae-myriantha* and *N. montana*.
