# Supplementary material for: Is the Co-Occurrence of Neophysopella meliosmae-myrianthae and N. montana (Pucciniales) Common on Grapevines in Japan?
Source: J Fungi (Basel). 2025 Mar 3;11(3):193. doi: 10.3390/jof11030193 (PMC11943129; doi:10.3390/jof11030193)
Supplement: Supplementary file 1 [file jof-11-00193-s001.zip › jof-3415858-supplementary/Supplementary Figure S2.pdf]

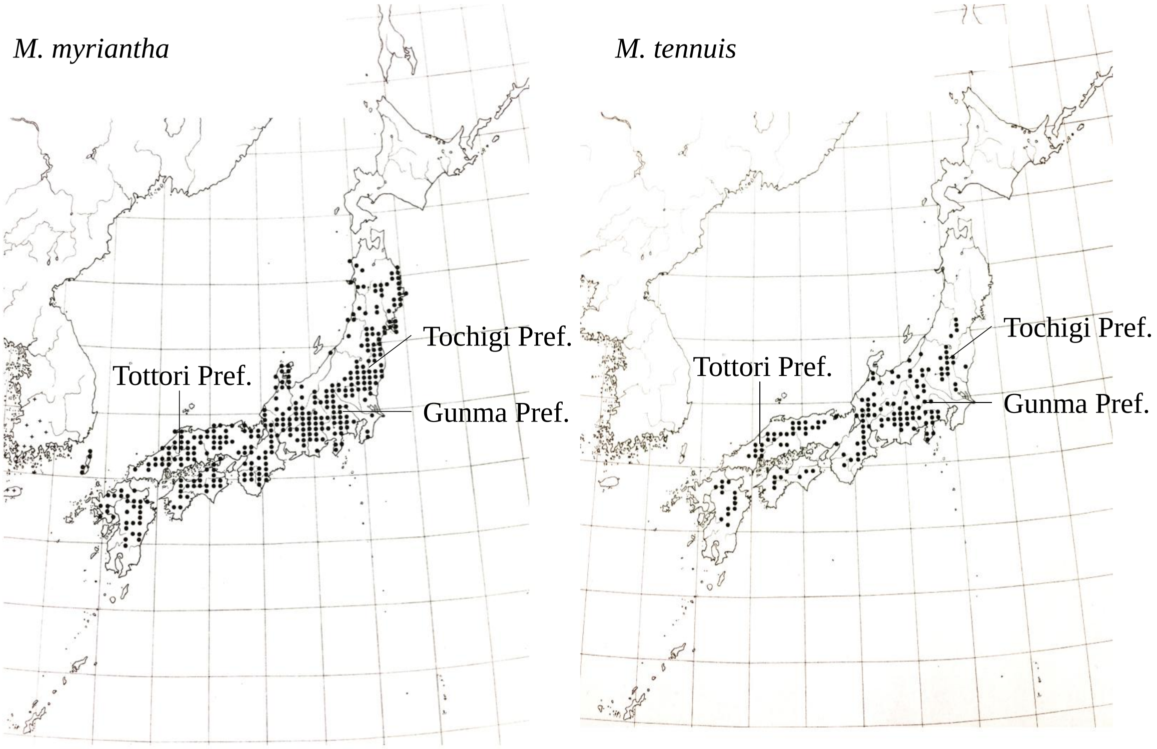

Supplementary Figure S1. Distribution map of *Meliosma myriantha* (left) and *M. tenuis* (right) in Japan (Horikawa, 1972)
