# Supplementary material for: Is the Co-Occurrence of Neophysopella meliosmae-myrianthae and N. montana (Pucciniales) Common on Grapevines in Japan?
Source: J Fungi (Basel). 2025 Mar 3;11(3):193. doi: 10.3390/jof11030193 (PMC11943129; doi:10.3390/jof11030193)
Supplement: Supplementary file 1 [file jof-11-00193-s001.zip › jof-3415858-supplementary/Supplementary Table S1.pdf]

Supplementary Table S1. List of location, geographical coordinate, and collection date of cultivated grapevine samples tested.

Specimen number	Location	Geographical coordinates (LAT, LNG)	Collection date
TSH-R58051 (= IBAR 10118)	Arakawa, Chichibu City, Saitama Prefecture	35.9574801, 139.0105053	Sep. 12, 2009
TSH-R58052 (= IBAR 10119)	Arakawa, Chichibu City, Saitama Prefecture	35.9574801, 139.0105053	Sep. 12, 2009
TSH-R58171 (= IBAR 10241)	Hiroshima Agricultural Technology Center, Higashihiroshima City, Hiroshima Prefecture	34.4196298, 132.6991500	Nov. 27, 2009
TSH-R58172 (= IBAR 10242)	Hiroshima Agricultural Technology Center, Higashihiroshima City, Hiroshima Prefecture	34.4196298, 132.6991500	Nov. 27, 2009
TSH-R58387 (= IBAR 10466)	Hojo, Hokuei Town, Tohaku, Tottori Prefecture	35.4788586, 133.8220927	Sep. 24, 2013
TSH-R58388 (= IBAR 10467)	Kose, Mimasaka City, Okayama Prefecture	34.969107, 134.1387934	Sep. 25, 2013
TSH-R58389 (= IBAR 10468)	Kose, Mimasaka City, Okayama Prefecture	34.969107, 134.1387934	Sep. 25, 2013
TSH-R58390 (= IBAR 10469)	Kose, Mimasaka City, Okayama Prefecture	34.969107, 134.1387934	Sep. 25, 2013
TSH-R58391 (= IBAR 10470)	Kose, Mimasaka City, Okayama Prefecture	34.969107, 134.1387934	Sep. 25, 2013
TSH-R58392 (= IBAR 10471)	Saeki Town, Wake, Okayama Prefecture	34.8487424, 134.0924659	Sep. 25, 2013
TSH-R58393 (= IBAR 10472)	Saeki Town, Wake, Okayama Prefecture	34.8487424, 134.0924659	Sep. 25, 2013
TSH-R58394 (= IBAR 10473)	Saeki Town, Wake, Okayama Prefecture	34.8487424, 134.0924659	Sep. 25, 2013
TSH-R58395 (= IBAR 10474)	Saeki Town, Wake, Okayama Prefecture	34.8487424, 134.0924659	Sep. 25, 2013
TSH-R58397 (= IBAR 10476)	National Route 429, Kita City, Okayama Prefecture	34.7834165, 133.7938674	Sep. 26, 2013
TSH-R58398 (= IBAR 10477)	Prefectural Route 66, Katta, Maniwa City, Okayama Prefecture	34.9852388, 133.7286194	Sep. 26, 2013
TSH-R58399 (= IBAR 10478)	Yoshikawa, Kibichuo Town, Kaga, Okayama Prefecture	34.8222293, 133.7429863	Sep. 26, 2013
TSH-R58400 (= IBAR 10479)	Yoshikawa, Kibichuo Town, Kaga, Okayama Prefecture	34.8222293, 133.7429863	Sep. 26, 2013
TSH-R58401 (= IBAR 10480)	Yoshikawa, Kibichuo Town, Kaga, Okayama Prefecture	34.8222293, 133.7429863	Sep. 26, 2013
TSH-R58402 (= IBAR 10481)	Shirochi, Ochiai Town, Takahashi City, Okayama Prefecture	34.7981225, 133.5462744	Sep. 26, 2013
TSH-R58403 (= IBAR 10482)	Higashikarube, Akaiwa City, Okayama Prefecture	34.8036866, 134.0270562	Sep. 26, 2013
TSH-R58404 (= IBAR 10483)	Higashikarube, Akaiwa City, Okayama Prefecture	34.8036866, 134.0270562	Sep. 26, 2013
TSH-R58405 (= IBAR 10484)	Ashigakubo, Yokoze Town, Chichibu, Saitama Prefecture	35.9761815, 139.1524819	Oct. 11, 2013
TSH-R58406 (= IBAR 10485)	Ashigakubo, Yokoze Town, Chichibu, Saitama Prefecture	35.9761815, 139.1524819	Oct. 11, 2013
TSH-R58407 (= IBAR 10486)	Ashigakubo, Yokoze Town, Chichibu, Saitama Prefecture	35.9761815, 139.1524819	Oct. 11, 2013
TSH-R58408 (= IBAR 10487)	Yokoze, Yokoze Town, Chichibu, Saitama Prefecture	35.9862973, 139.1026332	Oct. 11, 2013
TSH-R58409 (= IBAR 10488)	Makiokacho Kurashina, Yamanashi City, Yamanashi Prefecture	35.7583155, 138.686525	Oct. 11, 2013
TSH-R58410 (= IBAR 10489)	Higashi, Yamanashi City, Yamanashi Prefecture	35.7125337, 138.6965591	Oct. 11, 2013
TSH-R58411 (= IBAR 10490)	Ochiai, Yamanashi City, Yamanashi Prefecture	35.6972914, 138.6627013	Oct. 11, 2013
TSH-R58412 (= IBAR 10491)	Kasugai, Fuefuki City, Yamanashi Prefecture	35.6602064, 138.6598155	Oct. 11, 2013
TSH-R58413 (= IBAR 10492)	Sakurai, Fuefuki City, Yamanashi Prefecture	35.6630088, 138.6213476	Oct. 11, 2013
TSH-R58414 (= IBAR 10493)	Isawa Town, Fuefuki City, Yamanashi Prefecture	35.6492664, 138.6401409	Oct. 11, 2013
TSH-R58415 (= IBAR 10494)	Enzan Kaminishi, Koshu City, Yamanashi Prefecture	35.7054135, 138.7346248	Oct. 12, 2013
TSH-R58416 (= IBAR 10495)	Enzan Kamioso, Koshu City, Yamanashi Prefecture	35.7075038, 138.7254171	Oct. 12, 2013
TSH-R58417 (= IBAR 10496)	Yama, Katsunuma Town, Koshu City, Yamanashi Prefecture	35.6635144, 138.7316925	Oct. 12, 2013
TSH-R58418 (= IBAR 10497)	Osade, Katsunuma Town, Koshu City, Yamanashi Prefecture	35.6744537, 138.7279272	Oct. 12, 2013
TSH-R58419 (= IBAR 10498)	Katsunuma, Katsunuma Town, Koshu City, Yamanashi Prefecture	35.6635144, 138.7316925	Oct. 12, 2013
TSH-R58420 (= IBAR 10499)	Katsunuma, Katsunuma Town, Koshu City, Yamanashi Prefecture	35.6635144, 138.7316925	Oct. 12, 2013
TSH-R58421 (= IBAR 10500)	Kamiyama Town, Nirasaki City, Yamanashi Prefecture	35.7038151, 138.4262736	Oct. 12, 2013
TSH-R58422 (= IBAR 10501)	Kamiyama Town, Nirasaki City, Yamanashi Prefecture	35.7038151, 138.4262736	Oct. 12, 2013
TSH-R58427 (= IBAR 10506)	Kamishiroi, Shibukawa City, Gunma Prefecture	36.5628189, 139.0261692	Oct. 14, 2013
TSH-R58430 (= IBAR 10509)	Shimotsubara, Iwafune Town, Shimotsuga, Tochigi Prefecture	36.3173961, 139.6381969	Oct. 14, 2013
TSH-R58431 (= IBAR 10510)	Shimotsubara, Iwafune Town, Shimotsuga, Tochigi Prefecture	36.3173961, 139.6381969	Oct. 14, 2013
TSH-R58432 (= IBAR 10511)	Shimotsubara, Iwafune Town, Shimotsuga, Tochigi Prefecture	36.3173961, 139.6381969	Oct. 14, 2013
TSH-R58433 (= IBAR 10512)	Nishiyamada, Oohira Town, Tochigi City, Tochigi Prefecture	36.3490366, 139.6758241	Oct. 14, 2013
TSH-R58434 (= IBAR 10513)	Nishiyamada, Oohira Town, Tochigi City, Tochigi Prefecture	36.3490366, 139.6758241	Oct. 14, 2013
TSH-R58435 (= IBAR 10514)	Nishiyamada, Oohira Town, Tochigi City, Tochigi Prefecture	36.3490366, 139.6758241	Oct. 14, 2013
TSH-R30450	Ezohara, Yamanashi City, Yamanashi Prefecture	35.7026981, 138.6702235	Oct. 19, 2015
TSH-R30451	Nanokaichiba, Yamanashi City, Yamanashi Prefecture	35.7055355, 138.7015768	Oct. 19, 2015
TSH-R30453	Oyama, Misaka Town, Fuefuki City, Yamanashi Prefecture	35.6178157, 138.6764928	Oct. 18, 2015
TSH-R30455	Katsunuma Town, Koshu City, Yamanashi Prefecture	35.6635144, 138.7316925	Oct. 18, 2015
TSH-R30456	Nanokaichiba, Yamanashi City, Yamanashi Prefecture	35.6929987, 138.6714773	Oct. 18, 2015
TSH-R30457	Manriki, Yamanashi City, Yamanashi Prefecture	35.6929987, 138.6714773	Oct. 16, 2015
TSH-R30461	Shimoidai Town, Matsuyama City, Ehime Prefecture	33.8767456, 132.7988727	Oct. 16, 2015
TSH-R30462	Shimoidai Town, Matsuyama City, Ehime Prefecture	33.8767456, 132.7988727	Oct. 16, 2015
TSH-R30463	Fruit-Tree Experiment Station, Tenno, Katagami City, Akita Prefecture	39.8590503, 140.01733	2015
TSH-R30464	Fruit-Tree Experiment Station, Tenno, Katagami City, Akita Prefecture	39.8590503, 140.01733	2015
TSH-R30466	Kuroki, Soma City, Fukushima Prefecture	37.8109744, 140.9002806	Oct. 6, 2015
TSH-R30467	Yokone Town, Kofu City, Yamanashi Prefecture	35.6577424, 138.6099011	Oct. 6, 2015
TSH-R30468	Yokone Town, Kofu City, Yamanashi Prefecture	35.6577424, 138.6099011	Oct. 6, 2015
TSH-R30469	Yokone Town, Kofu City, Yamanashi Prefecture	35.6577424, 138.6099011	Oct. 5, 2015
TSH-R30470	Pre. Res. Ins. for the Environ., Agricul., For. and Fisher., Habikino City, Osaka Prefecture	34.535217, 135.5985176	Sep. 9, 2015
TSH-R30472	Akaiwa City, Okayama Prefecture	34.7553712, 134.0187579	Sep. 11, 2015
TSH-R30473	Aono, Ibara City, Okayama Prefecture	34.6313395, 133.4717027	Sep. 11, 2015
TSH-R30475	Tsuchida, Okayama City, Okayama Prefecture	34.6963288, 133.9853855	Sep. 11, 2015
TSH-R30476	Nagasaki, Funao Town, Kurashiki City, Okayama Prefecture	32.7503334, 129.8778888	Sep. 11, 2015
TSH-R30477	Izumo City, Shimane Prefecture	35.3670442, 132.7546943	Sep. 24, 2015
HHUF 10428	Sakaimatsu, Kuroishi City, Aomori Prefecture	40.6540265, 140.5710457	Nov. 6, 1979
HHUF 15175	Oohasama Town, Hanamaki City, Iwate Prefecture	39.479065, 141.2898537	Sep. 19, 1984
TSH-R30478	Hashimoto, Yazu Town, Yazu, Tottori Prefecture	35.3486654, 134.2455322	Nov. 3, 2016
TSH-R30517	Atsugi City, Kanagawa Prefecture	35.44309399, 139.3625403	Sep. 4, 2015
TSH-R30480	Kita Town, Kobe City, Hyogo Prefecture	34.8332544, 135.2192058	Sep. 12, 2015
TSH-R30482	Kyoto City, Kyoto Prefecture	35.011564, 135.7681489	Sep. 13, 2015
TSH-R30483	Kyoto City, Kyoto Prefecture	35.011564, 135.7681489	Sep. 13, 2015
TSH-R30484	Kyoto City, Kyoto Prefecture	35.011564, 135.7681489	Sep. 13, 2015
TSH-R30485	Kyoto City, Kyoto Prefecture	35.011564, 135.7681489	Sep. 13, 2015
TSH-R30487	Ryuo Town, Shiga Prefecture	35.0607672, 136.1244806	Sep. 13, 2015
TSH-R30488	Ryuo Town, Shiga Prefecture	35.0607672, 136.1244806	Sep. 13, 2015
TSH-R30489	Nabari City, Mie Prefecture	34.6275071, 136.1084508	Sep. 14, 2015
TSH-R30491	Kashiwara City, Osaka Prefecture	34.5791141, 135.6283141	Sep. 14, 2015
TSH-R30493	Aridagawa Town, Wakayama Prefecture	34.0704634, 135.2298848	Sep. 15, 2015

TSH-R30495	Okaya City, Nagano Prefecture	36.0670021, 138.0493709	Sep. 21, 2015
TSH-R30496	Okaya City, Nagano Prefecture	36.0670021, 138.0493709	Sep. 21, 2015
TSH-R30497	Ikusaka Village, Higashichikuma, Nagano Prefecture	36.42517, 137.9274909	Sep. 26, 2015
TSH-R30499	Ikusaka Village, Higashichikuma, Nagano Prefecture	36.42517, 137.9274909	Sep. 26, 2015
TSH-R30505	Kasumigaura City, Ibaraki Prefecture	36.0942581, 140.3307847	Oct. 30, 2015
TSH-R30507	Kasumigaura City, Ibaraki Prefecture	36.0942581, 140.3307847	Oct. 30, 2015
TSH-R30515	Kasumigaura City, Ibaraki Prefecture	36.0942581, 140.3307847	Oct. 30, 2015
TSH-R30516	Kasumigaura City, Ibaraki Prefecture	36.0942581, 140.3307847	Oct. 30, 2015
TSH-R30518	Tokyo Univ. of Agricul. and Tech. Isehara Farm, Sannomiya, Isehara City, Kanagawa Prefecture	35.4011378, 139.2819528	Nov. 4, 2015
TSH-R30519	Tokyo Univ. of Agricul. and Tech. Isehara Farm, Sannomiya, Isehara City, Kanagawa Prefecture	35.4011378, 139.2819528	Nov. 4, 2015
TSH-R30520	Tokyo Univ. of Agricul. and Tech. Isehara Farm, Sannomiya, Isehara City, Kanagawa Prefecture	35.4011378, 139.2819528	Nov. 4, 2015
TSH-R30521	Kaminoyama City, Yamagata Prefecture	38.1495569, 140.2678429	Nov. 13, 2015
TSH-R30522	Kaminoyama City, Yamagata Prefecture	38.1495569, 140.2678429	Nov. 13, 2015
TSH-R30523	Kaminoyama City, Yamagata Prefecture	38.1495569, 140.2678429	Nov. 13, 2015
TSH-R30526	Kaminoyama City, Yamagata Prefecture	38.1495569, 140.2678429	Nov. 13, 2015
TSH-R30527	Kaminoyama City, Yamagata Prefecture	38.1495569, 140.2678429	Nov. 13, 2015
TSH-R30530	Kaminoyama City, Yamagata Prefecture	38.1495569, 140.2678429	Nov. 13, 2015
TSH-R30531	Nihonmatsu City, Fukushima Prefecture	37.5848714, 140.4312591	Nov. 4, 2016
TSH-R30532	Nihonmatsu City, Fukushima Prefecture	37.5848714, 140.4312591	Nov. 4, 2016
TSH-R30533	Nihonmatsu City, Fukushima Prefecture	37.5848714, 140.4312591	Nov. 4, 2016
TSH-R30534	Nihonmatsu City, Fukushima Prefecture	37.5848714, 140.4312591	Nov. 4, 2016
TSH-R30486	Kyoto City, Kyoto Prefecture	35.011564, 135.7681489	Sep. 16, 2016
TSH-R30543	Takamatsu City, Kagawa Prefecture	34.342542, 134.0465405	Sep. 9, 2016

HHUF: The Mycological Herbarium of Hirosaki University, IBAR: The Herbarium of Systematic Mycology, Ibaraki University, TSH-R: The Rust collection of Mycological Herbarium of the University of Tsukuba.
