# Supplementary material for: Is the Co-Occurrence of Neophysopella meliosmae-myrianthae and N. montana (Pucciniales) Common on Grapevines in Japan?
Source: J Fungi (Basel). 2025 Mar 3;11(3):193. doi: 10.3390/jof11030193 (PMC11943129; doi:10.3390/jof11030193)
Supplement: Supplementary file 1 [file jof-11-00193-s001.zip › jof-3415858-supplementary/Supplementary Table S2.pdf]

Supplementary Table S2. List of locality, geographical coordinate, and collection date of wild grapevine, *Vitis coignetiae* , samples tested.

Specimen number	Location	Geographical coordinates (LAT, LNG)	Collection date
TSH-R51432 (= IBAR 1817)	Mt. Owasezawasan, Yama, Fukushima Prefecture	37.7191226, 140.0975877	Sep. 28, 1979
TSH-R52161 (= IBAR 2782)	Takizawa forest road, Mt. Fuji, Yamanashi Prefecture	35.3616731, 138.7271056	Sep. 6, 1983
TSH-R52162 (= IBAR 2783)	Takizawa forest road, Mt. Fuji, Yamanashi Prefecture	35.3616731, 138.7271056	Sep. 6, 1983
TSH-R53179 (= IBAR 3930)	Nasu Town, Nasu, Tochigi Prefecture	37.0197825, 140.1209869	Oct. 1, 1989
TSH-R54801 (= IBAR 6279)	Mt. Yahikoyama, Yahiko Village, Nishikanbara, Niigata Prefecture	37.7046682, 138.809529	Oct. 5, 1992
TSH-R54804 (= IBAR 6282)	Mt. Yahikoyama, Yahiko Village, Nishikanbara, Niigata Prefecture	37.7046682, 138.809529	Oct. 5, 1992
TSH-R58370 (= IBAR 10449)	Nakamiyori, Nikko City, Tochigi Prefecture	37.0264862, 139.6809281	Sep. 9, 2013
TSH-R58371 (= IBAR 10450)	Yunishigawa, Nikko City, Tochigi Prefecture	36.9799529, 139.5891233	Sep. 9, 2013
TSH-R58372 (= IBAR 10451)	Yunishigawa, Nikko City, Tochigi Prefecture	36.9799529, 139.5891233	Sep. 9, 2013
TSH-R58373 (= IBAR 10452)	Yunishigawa, Nikko City, Tochigi Prefecture	36.9799529, 139.5891233	Sep. 9, 2013
TSH-R58374 (= IBAR 10453)	Yunishigawa, Nikko City, Tochigi Prefecture	36.9799529, 139.5891233	Sep. 9, 2013
TSH-R58375 (= IBAR 10454)	Yunishigawa, Nikko City, Tochigi Prefecture	36.9799529, 139.5891233	Sep. 9, 2013
TSH-R58376 (= IBAR 10455)	Yunishigawa, Nikko City, Tochigi Prefecture	36.9799529, 139.5891233	Sep. 9, 2013
TSH-R58377 (= IBAR 10456)	Yasugamori Forest Road, Minamiaizu, Fukushima Prefecture	37.0162502, 139.6087071	Sep. 9, 2013
TSH-R58378 (= IBAR 10457)	Yasugamori Forest Road, Minamiaizu, Fukushima Prefecture	37.0162502, 139.6087071	Sep. 9, 2013
TSH-R58379 (= IBAR 10458)	Mt. Daisen, Daisen Town, Saihaku, Tottori Prefecture	35.3796267, 133.5462744	Sep. 24, 2013
TSH-R58380 (= IBAR 10459)	Mt. Daisen, Daisen Town, Saihaku, Tottori Prefecture	35.3796267, 133.5462744	Sep. 24, 2013
TSH-R58381 (= IBAR 10460)	Mt. Daisen, Daisen Town, Saihaku, Tottori Prefecture	35.3796267, 133.5462744	Sep. 24, 2013
TSH-R58384 (= IBAR 10463)	Mt. Daisen, Daisen Town, Saihaku, Tottori Prefecture	35.3796267, 133.5462744	Sep. 24, 2013
TSH-R58385 (= IBAR 10464)	Mt. Daisen, Daisen Town, Saihaku, Tottori Prefecture	35.3796267, 133.5462744	Sep. 24, 2013
TSH-R58386 (= IBAR 10465)	Okudaisen, Kofu Town, Hino, Tottori Prefecture	35.2957635, 133.4741754	Sep. 24, 2013
TSH-R58428 (= IBAR 10507)	Mt. Omineyama, Minakami Town, Tone, Gunma Prefecture	36.7356059, 138.9341265	Oct. 14, 2013
TSH-R58429 (= IBAR 10508)	Mt. Omineyama, Minakami Town, Tone, Gunma Prefecture	36.7356059, 138.9341265	Oct. 14, 2013
HHUF 3963	Hengasa Forest Road, Hirosaki City, Aomori Prefecture	40.6306055, 140.4562442	Sep. 23, 1972
HHUF 11069	Jizotai, Gonohe Town, Sannohe, Aomori Prefecture	40.5082609, 141.3261055	Sep. 9, 1980
TSH-R30536	Mt. Daisen, Daisen Town, Saihaku, Tottori Prefecture	35.3796267, 133.5462744	Sep. 12, 2015
TSH-R30537	Mt. Daisen, Daisen Town, Saihaku, Tottori Prefecture	35.3796267, 133.5462744	Sep. 12, 2015
TSH-R30538	Mt. Daisen, Daisen Town, Saihaku, Tottori Prefecture	35.3796267, 133.5462744	Sep. 12, 2015
TSH-R30539	Mt. Daisen, Daisen Town, Saihaku, Tottori Prefecture	35.3796267, 133.5462744	Sep. 12, 2015
TSH-R30540	Mt. Daisen, Daisen Town, Saihaku, Tottori Prefecture	35.3796267, 133.5462744	Sep. 12, 2015
TSH-R30541	Mt. Daisen, Daisen Town, Saihaku, Tottori Prefecture	35.3796267, 133.5462744	Sep. 12, 2015
TSH-R30542	Mt. Daisen, Daisen Town, Saihaku, Tottori Prefecture	35.3796267, 133.5462744	Sep. 12, 2015
TSH-R2204	Nobeyama, Minamimaki Village, Minamisaku, Nagano Prefecture	35.9451607, 138.4696874	Oct. 8, 1968
TSH-R2205	Nobeyama, Minamimaki Village, Minamisaku, Nagano Prefecture	35.9451607, 138.4696874	Oct. 8, 1968
TSH-R2206	Nobeyama, Minamimaki Village, Minamisaku, Nagano Prefecture	35.9451607, 138.4696874	Oct. 8, 1968
TSH-R2207	Nobeyama, Minamimaki Village, Minamisaku, Nagano Prefecture	35.9451607, 138.4696874	Sep. 3, 1975
TSH-R2803	Nobeyama, Minamimaki Village, Minamisaku, Nagano Prefecture	35.9451607, 138.4696874	Oct. 8, 1968
TSH-R2804	Nobeyama, Minamimaki Village, Minamisaku, Nagano Prefecture	35.9451607, 138.4696874	Oct. 8, 1968
TSH-R2805	Nobeyama, Minamimaki Village, Minamisaku, Nagano Prefecture	35.9451607, 138.4696874	Oct. 8, 1968
TSH-R2806	Nobeyama, Minamimaki Village, Minamisaku, Nagano Prefecture	35.9451607, 138.4696874	Oct. 8, 1968
TSH-R3452	Nobeyama, Minamimaki Village, Minamisaku, Nagano Prefecture	35.9451607, 138.4696874	Sep. 6, 1967
TSH-R13225	Lake Yamanakako, Yamanashi Prefecture	35.4106457, 138.8611439	Oct. 9, 1969
TSH-R1606	Niigata Prefecture	36.8509801, 138.8466961	Sep. 5, 1987
TSH-R30544	Nobeyama, Minamimaki Village, Minamisaku, Nagano Prefecture	35.9451607, 138.4696874	Sep. 19, 2024

HHUF: The Mycological Herbarium of Hirosaki University, IBAR: The Herbarium of Systematic Mycology, Ibaraki University, TSH-R: The Rust collection of Mycological Herbarium of the University of Tsukuba.
